# Multicystic peritoneal mesothelioma mimicking recurrent pseudomyxoma peritonei

**DOI:** 10.1093/jscr/rjag525

**Published:** 2026-07-07

**Authors:** Alessia Scarton, Marco Tonello, Elisa Pizzolato, Carola Cenzi, Ivana Cataldo, Vasileios Mourmouras, Pierluigi Pilati, Antonio Sommariva

**Affiliations:** Surgical Oncology Department, Unit of Surgical Oncology of Digestive Tract, Veneto Institute of Oncology IOV-IRCCS, Padua, Italy; Surgical Oncology Department, Unit of Surgical Oncology of Digestive Tract, Veneto Institute of Oncology IOV-IRCCS, Padua, Italy; Surgical Oncology Department, Unit of Surgical Oncology of Digestive Tract, Veneto Institute of Oncology IOV-IRCCS, Padua, Italy; Clinical Research Unit, Veneto Institute of Oncology IOV-IRCCS, Padua, Italy; Surgical Pathology Unit, Veneto Institute of Oncology IOV-IRCCS, Padua, Italy; Surgical Pathology Unit, Veneto Institute of Oncology IOV-IRCCS, Padua, Italy; Surgical Oncology Department, Unit of Surgical Oncology of Digestive Tract, Veneto Institute of Oncology IOV-IRCCS, Padua, Italy; Surgical Oncology Department, Unit of Surgical Oncology of Digestive Tract, Veneto Institute of Oncology IOV-IRCCS, Padua, Italy

**Keywords:** multicystic peritoneal mesothelioma, peritoneal pseudomyxoma, laparoscopic cytoreductive surgery, HIPEC

## Abstract

Multicystic peritoneal mesothelioma (MCPM) is a borderline variant of peritoneal mesothelioma with indolent behaviour but a significant recurrence rate without radical treatment. Due to its rarity, risk factors and optimal treatment are still to clarify. The present clinical case is a report of MCPM developed after laparoscopic cytoreductive surgery and hyperthermic intraperitoneal chemotherapy (Lap CRS-HIPEC) for pseudomyxoma peritonei (PMP) of appendiceal origin. The patient was treated for PMP with Lap CRS-HIPEC. One year later a recurrence was suspected at the thorax-abdomen computed tomography scan. After multidisciplinary discussion, she underwent iterative Lap CRS-HIPEC. Pathological examinations revealed MCPM. After 3 years of follow-up the patient is disease-free for both tumours. This is a unique case of MCPM mimicking recurrent PMP. Lap CRS-HIPEC is an effective approach for both conditions.

## Introduction

Peritoneal mesotheliomas are rare malignancies exhibiting a wide range of clinical manifestations and levels of aggressiveness. Among the histological subtypes, multicystic peritoneal mesothelioma (MCPM) is typically regarded as a borderline form due to the high recurrence rate [1]. The report presents a case of a middle-aged woman diagnosed with MCPM 1 year following cytoreductive surgery (CRS) combined with hyperthermic intraperitoneal chemotherapy (HIPEC) for pseudomyxoma peritonei (PMP).

## Case presentation

A 56-years-old woman was referred to our outpatient clinic after an open splenectomy. The histopathological examination of the specimen revealed a low-grade PMP. Post-operative abdomen computed tomography (CT)-scan confirmed the presence of left sub-diaphragmatic peripancreatic mucin deposits and a minimal free fluid in the pelvis. The patient’s medical history included a quadrantectomy for oestrogen receptor-positive ductal breast cancer, followed by radiotherapy and a 5-year course of Tamoxifen. Additionally, she had undergone bilateral ovariectomy for a benign ovarian cyst and a lung wedge resection for a benign lump.

**Figure 1:**
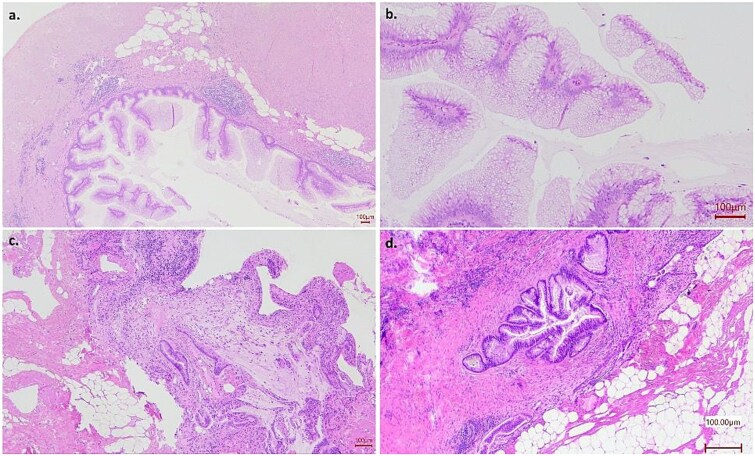
Low grade appendiceal mucinous tumour (LAMN) with pseudomyxoma peritonei grade 2. (a) Appendiceal fundus showing the presence of villous epithelial mucinous proliferation with low grade cytological atypia (b); pseudomyxoma peritonei of grade 2, showing peritoneal localization of low grade neoplastic mucinous epithelium with infiltrative pattern and moderate hypercellularity (c, d).

After a multidisciplinary discussion, CRS-HIPEC was proposed. Preoperative investigation was completed with Upper GI endoscopy, colonoscopy, and abdominal magnetic resonance imaging (MRI). Laparoscopic CRS (Lap CRS-HIPEC) was performed including total omentectomy, appendicectomy, excision of small nodules of the mesosigma and right subdiaphragmatic peritonectomy. HIPEC regimen was Mitomycin C and Cisplatin administered for 60 minutes at target temperature of 41°C. The peritoneal cancer index (PCI) was 4. Histopathological examination confirmed the presence of PMP (grade 2) associated with a low grade appendiceal mucinous neoplasm (LAMN) (Fig. 1). The postoperative course was uneventful, and the patient was discharged 6 days after the CRS-HIPEC. Regular follow-up was planned with clinical examination every 3 months and thorax-abdomen CT scan every 6 months.

**Figure 2:**
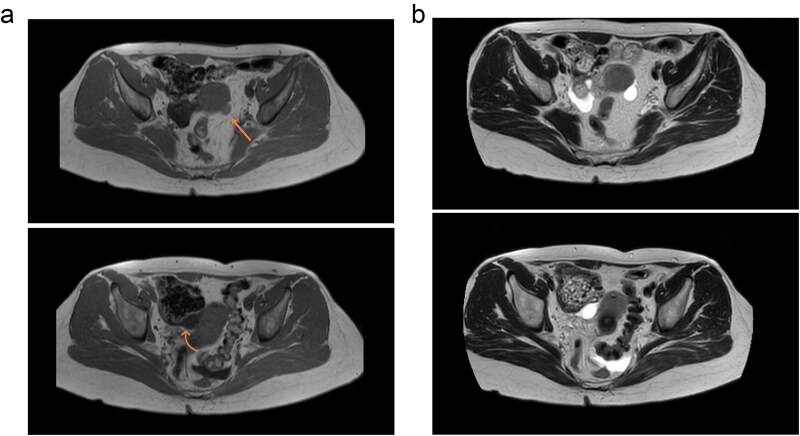
(a) Cystic lesions in T1-weightened MR images. Straight arrow indicates the cyst with haemorrhagic content, slightly hyperintense, whereas curved arrow the simple one. (b) Cystic lesions in T2-weightened MR images. In this sequence, the lesions are hyperintense.

**Figure 3:**
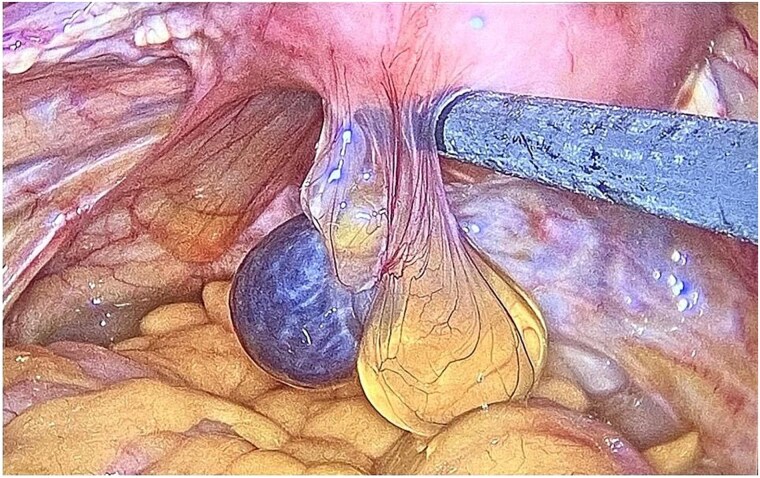
Intraoperative findings: Multiple cysts in pelvis with free fluid.

**Figure 4:**
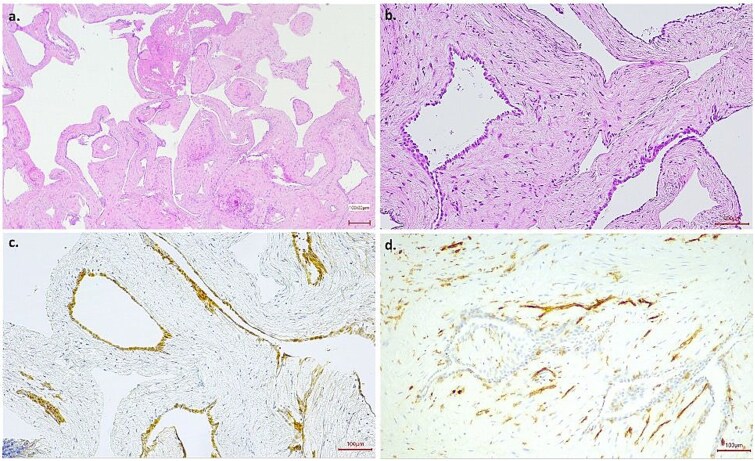
Multicystic peritoneal mesothelioma (i.e. multilocular peritoneal inclusion cyst, sec WHO 5th ed). (a) A multicystic proliferation with fibrous stroma; no infiltrative pattern nor stromal atypia was detected; (b) cysts were lined by flat-cuboidal epithelium without atypia which stained positive for calretinin (c) and negative for CD31 (lymphatic vessels as positive internal control) (d).

After almost 1 year the CT showed pelvic free fluid and pelvic plurilocular cysts suspected for disease recurrence. An abdominal MRI confirmed the findings (Fig. 2). After multidisciplinary discussion, an exploratory laparoscopy was proposed with cytoreduction and HIPEC if the disease recurrence was confirmed. The abdominal exploration confirmed the presence of multiloculated grape-like, fluid-containing cysts (Fig. 3) contiguous to the uterus and the cecum. No mucin was found. A complete resection was achieved with a fully laparoscopic approach. The intraoperative histological examination of the lesions was not conclusive for PMP. However, given the macroscopic appearance of the findings and the patient’s medical history, HIPEC with Cisplatin and Mitomycin C for 60 minutes was performed. No complications occurred and the patient was discharged after 5 days. The final histopathological examination revealed a multicystic lesion lined by flat-cuboidal epithelium without cytological atypia. Immunohistochemical analysis supported the mesothelial nature of the epithelial component which stained positive for calretinin and resulted negative for Podoplanin, CD31 and CDX2. Overall, the histopathological findings supported the diagnosis of MCPM (i.e. Multilocular peritoneal inclusion cyst, sec WHO 5th ed [2]) (Fig. 4).

## Discussion

MCPM has traditionally been considered a benign mesothelioma, and different theories have been proposed regarding its aetiology, with some authors suggesting an association with chronic peritoneal inflammation, such as that seen in conditions like endometriosis, previous abdominal surgeries, pelvic inflammatory disease [3], and familial Mediterranean fever [4]. Despite its slow growing nature, MCPM is associated with recurrence up to 50% after debulking surgery [5], and instances of malignant transformation have been described [6].

Mostly asymptomatic, patients with MCPM could complain vague abdominal pain or distensions, due to the progressive growth of the lesions. Urinary outflow obstruction or palpable mass have been reported [3]. MCPM appears as multiple grape-like cysts, from a few millimetres to over 20 cm, filled with clear serous fluid [7]. Comprehensive histopathologic evaluation by an expert pathologist is required to confirm the diagnosis and exclude differential diagnoses such as malignant mesothelioma, benign cystic lesions, lymphangioma, and endometriosis [8]. MCPM immunochemistry is usually positive for markers as Calretinin, WT-1, CK7, and D2–40 and variably for oestrogen and progesterone receptors [9]. Both Ki-67 and mitotic activity are low in MCPM when compared with the malignant counterpart and it could be explained by its pattern of growth [1]. Imaging could be helpful for differential diagnosis and both CT and MRI could provide information about the cysts, the extension, and the relationship with the surrounding organs, especially if they embed them. Lesions appear as multiple, most often located into the pelvis [10]. On MRI they appear as hypointense on T1 weighted images and mild to hyperintense in T2, although it may be the opposite in the case of cysts with a bloody content [3, 10].

Similar behaviour between MCMP and PMP—indolent development and a non-infiltrative pattern—suggests that the optimal treatment would be CRS-HIPEC [6]. Several studies had demonstrated excellent survival outcomes primarily using Cisplatin and Doxorubicin [7]. Firstly, Yan *et al* showed the impact of adding HIPEC to a complete CRS on disease control in a twelve patients’ multicentre study [5]. The study by Kepenekien *et al* [11] also showed that HIPEC was linked to a reduced incidence of recurrence and Nizri *et al* reported an 80% rate of disease-free survival at 10 years [12]. Reported recurrence rate ranges from 4% to 40%. The high recurrence rate and the potential for malignant transformation justify the aggressive treatment, despite ongoing debates regarding the neoplastic nature of the disease [8].

Lap CRS-HIPEC is a less invasive option to obtain a complete excision of peritoneal metastasis in selected cases [13]. The presented clinical vignette exemplifies an effective application of Lap CRS-HIPEC, both in PMP and MCPM, resulting in favourable postoperative outcome. The international PSOGI registry of Lap CRS-HIPEC reported nineteen cases of MCPM treated with this approach [14]. In a French study, 15 cases (25%) of Lap CRS-HIPEC for MCPM are reported. In this subgroup, median PCI was four and no recurrence was registered. No difference was shown regarding severe complications rate between open approach and Lap CRS-HIPEC, which was 27% and 7%, respectively [11].

## Conclusion

The reported clinical case is a unique scenario of MCPM presented after a diagnosis of PMP. The unusual history of this patient remarks again the similarity between the two diseases in radiological and intraoperative features. Previous surgeries and intraperitoneal chemotherapy may have triggered the development of MCPM.

## References

[1] Baratti D, Vaira M, Kusamura S et al. Multicystic peritoneal mesothelioma: outcomes and patho-biological features in a multi-institutional series treated by cytoreductive surgery and hyperthermic intraperitoneal chemotherapy (HIPEC). Eur J Surg Oncol 2010;36:1047–53. 10.1016/j.ejso.2010.08.130 20832234

[2] WHO Classification of Tumours Editorial Board. *Female Genital Tumours*. 5th ed. Vol. 4. Lyon (France): International Agency for Research on Cancer; 2020. ISBN-13 978-92-832-4504-9.

[3] Noiret B, Renaud F, Piessen G et al. Eveno C multicystic peritoneal mesothelioma: a systematic review of the literature. Pleura Peritoneum 2019;4:20190024. 10.1515/pp-2019-0024 31667333 PMC6812218

[4] Curgunlu A, Karter Y, Tüfekci IB et al. Benign cystic mesothelioma: a rare cause of ascites in a case with familial Mediterranean fever. Clin Exp Rheumatol 2003;21:S41–3. 14727459

[5] Yan TD, Deraco M, Baratti D et al. Cytoreductive surgery and hyperthermic intraperitoneal chemotherapy for malignant peritoneal mesothelioma: multi-institutional experience. J Clin Oncol 2009;27:6237–42. 10.1200/JCO.2009.23.9640 19917862

[6] Yan TD, Deraco M, Baratti D et al. Gilly FN malignant transformation of "benign" cystic mesothelioma of the peritoneum. J Surg Oncol 2002;79:243–51. 10.1002/jso.10081 11920782

[7] Chatterjee A, Bhatt A. Rare variants of malignant peritoneal mesothelioma: a literature review. Indian J Surg Oncol 2023;14:30–8. 10.1007/s13193-023-01754-4 37359922 PMC10284736

[8] Kusamura S, Kepenekian V, Villeneuve L et al. Peritoneal mesothelioma: PSOGI/EURACAN clinical practice guidelines for diagnosis, treatment and follow-up. Eur J Surg Oncol 2021;47:36–59. 10.1016/j.ejso.2020.02.011 32209311

[9] Kemp AM, Nayar R, De Frias D et al. Cytomorphologic characteristics of fine needle core biopsy of multicystic peritoneal mesothelioma: a case report and review of the literature. Diagn Cytopathol 2010;38:192–7. 10.1002/dc.21192 19813256

[10] Mehta V, Chowdhary V, Sharma R et al. Imaging appearance of benign multicystic peritoneal mesothelioma: a case report and review of the literature. Clin Imaging 2017;42:133–7. 10.1016/j.clinimag.2016.10.008 27984828

[11] Kepenekian V, Péron J, Goéré D et al. Multicystic peritoneal mesothelioma treated with cytoreductive surgery followed or not by hyperthermic intraperitoneal chemotherapy: results from a large multicentric cohort. Int J Hyperthermia 2021;38:805–14. 10.1080/02656736.2021.1903585 34039244

[12] Nizri E, Baratti D, Guaglio M et al. Multicystic mesothelioma: operative and long-term outcomes with cytoreductive surgery and hyperthermic intra peritoneal chemotherapy. Eur J Surg Oncol 2018;44:1100–4. 10.1016/j.ejso.2018.03.004 29703622

[13] Sommariva A, Tonello M, De Simoni O et al. Laparoscopic hyperthermic intraperitoneal chemotherapy for appendiceal tumors. Asian J Endosc Surg 2020;13:614–7. 10.1111/ases.12784 31997552

[14] Arjona-Sanchez A, Aziz O, Passot G et al. Laparoscopic cytoreductive surgery and hyperthermic intraperitoneal chemotherapy: long term oncologic outcomes from the international PSOGI registry. Eur J Surg Oncol 2023;49:107001. 10.1016/j.ejso.2023.107001 37579618

